# [Retracted] CALB1 enhances the interaction between p53 and MDM2, and inhibits the senescence of ovarian cancer cells

**DOI:** 10.3892/mmr.2026.14027

**Published:** 2026-09-21

**Authors:** Long-Qiao Cao, Ya-Nan Wang, Ming Liang, Mei-Zhou Pan

Mol Med Rep 19: 5097–5104, 2019; DOI: 10.3892/mmr.2019.10212

Following the publication of the above paper, it was drawn to the Editor's attention by a concerned reader that the p16 antibody that the authors reported to have used for the western blot experiment shown in Fig. 4B (cat. no. ab51243) is specific for the protein known as P16 ARC (or ARPC5), not the apoptosis-associated protein p16INK4a, as was intended. Furthermore, upon performing an independent analysis of the data in this paper in the Editorial Office, it came to light that western blot data featured in Fig. 5 were strikingly similar to data that had already been published in a different paper in the journal *Oncotarget* that featured no authors in common, and western blot data included in Fig. 1B were also very similar to data in another article, which again featured no authors in common, that was submitted around the same time to the journal *OncoTargets and Therapy*.

Owing to the fact that the contentious data in the above article had already been published prior to its submission to *Molecular Medicine Reports*, the Editor has decided that this paper should be retracted from the Journal. The authors were asked for an explanation to account for these concerns, but the Editorial Office did not receive a reply. The Editor apologizes to the readership for any inconvenience caused.

